# Pencil beam scanning dosimetry for large animal irradiation

**DOI:** 10.1093/jrr/rru029

**Published:** 2014-05-22

**Authors:** Liyong Lin, Timothy D. Solberg, Alexandro Carabe, James E. Mcdonough, Eric Diffenderfer, Jenine K. Sanzari, Ann R. Kennedy, Keith Cengel

**Affiliations:** Department of Radiation Oncology, University of Pennsylvania, 3400 Civic Center Blvd, 2326 TRC, PCAM, Philadelphia, PA 19104, USA

**Keywords:** pencil beam scanning, proton therapy, large animal, solar particle event, total body irradiation

## Abstract

The space radiation environment imposes increased dangers of exposure to ionizing radiation, particularly during a solar particle event. These events consist primarily of low-energy protons that produce a highly inhomogeneous depth–dose distribution. Here we describe a novel technique that uses pencil beam scanning at extended source-to-surface distances and range shifter (RS) to provide robust but easily modifiable delivery of simulated solar particle event radiation to large animals. Thorough characterization of spot profiles as a function of energy, distance and RS position is critical to accurate treatment planning. At 105 MeV, the spot sigma is 234 mm at 4800 mm from the isocentre when the RS is installed at the nozzle. With the energy increased to 220 MeV, the spot sigma is 66 mm. At a distance of 1200 mm from the isocentre, the Gaussian sigma is 68 mm and 23 mm at 105 MeV and 220 MeV, respectively, when the RS is located on the nozzle. At lower energies, the spot sigma exhibits large differences as a function of distance and RS position. Scan areas of 1400 mm (superior–inferior) by 940 mm (anterior–posterior) and 580 mm by 320 mm are achieved at the extended distances of 4800 mm and 1200 mm, respectively, with dose inhomogeneity <2%. To treat large animals with a more sophisticated dose distribution, spot size can be reduced by placing the RS closer than 70 mm to the surface of the animals, producing spot sigmas below 6 mm.

## INTRODUCTION

Outside of the protection afforded by the Earth's magnetosphere, exposure to charged particle radiation becomes a significant human health concern. The major sources of radiation exposure include galactic cosmic rays (GCRs), comprised primarily of high-energy, high-mass-number particles with a relatively constant background fluence rate, and solar particle event (SPE) radiation. SPE radiation is primarily composed of protons with energies ranging from MeV to GeV, heavily skewed in favour of <100 MeV protons [1–2]. As a consequence of this energy–fluence distribution, SPEs are predicted to produce a depth–dose distribution within an exposed astronaut that is highly inhomogeneous with a relatively high superficial dose and a lower internal dose [3].

Animal models are critical for studying toxicity and evaluating mitigators for radiation exposure. In the context of whole-body exposure to SPE protons, the energy–fluence distribution and the resultant inhomogeneous depth–dose distribution necessitate careful consideration of animal size [4–6]. In experimental models that are much smaller than humans (e.g. mice, rats and ferrets), simply scaling the proton energies to match the dose distribution dramatically alters the linear energy transfer (LET) spectrum for the protons. Conversely, delivering a simulated SPE to smaller animals without scaling the energies would match the LET spectrum of an SPE, but create an inhomogeneous dose distribution that is higher on the inside than outside, the exact inverse of the human SPE dose distribution. For larger animals (e.g. pigs), it is possible to match the anticipated dose distribution for a human SPE exposure using protons with a similar LET spectrum, but using double-scattered proton beams requires the time-consuming creation and testing of unique range/energy modulators to study the effect of specific energy–fluence/dose distributions. Scattered proton beams can also be problematic in large animal irradiation due to field size limitations at many facilities and lack of uniformity in the spreadout Bragg peaks at extended distances [7]. Recently, proton pencil beam scanning (PBS) systems have been developed and installed for clinical use in the USA and elsewhere [8–10]. The University of Pennsylvania has a dedicated PBS horizontal beam developed by Ion Beam Applications (Louvain-la-Neuve, Belgium) that is clinically commissioned for patient treatment. The general design of the system and description of spot characteristics have been described by Farr *et al*. [11]. Clasie *et al.* described the energy distribution and the Bragg peaks from the IBA Proteus Cyclotron [12]. Lin *et al.* has described the evolution of spot profiles in air and in phantom from the dedicated PBS horizontal beam [13]. Spot profiles are typically approximated with first order Gaussian function. Gaussian sigma and full-width half maximal (2.355 sigma) are typically reported for spot profiles. To accommodate large animals, we use the fixed horizontal beam with the source-to-surface distance (SSD) extended to 5 m and a range shifter (RS) to reproduce the <100 MeV portion of the SPE energy–fluence spectrum. While PBS can overcome the field size and uniformity limitations with extended SSD and RS, to date no detailed PBS dosimetry has been reported for irradiation across a wide range of experimental conditions.

In this study, the spot characteristics at extended SSD with the RS in different places along the beam axes are measured. Furthermore, we describe the development and testing of novel techniques to adapt PBS for use in large animal irradiation that are robust but easily modifiable to allow delivery of protons with a variety of energy–fluence distributions. Spots are energy-weighted to match the proton fractional depth dose (FDD) to that of mixed electron beams used previously in SPE experiments [4]. Measured beam profiles resulting from two scanning patterns at multiple energies are presented and compared with calculations from a commercial and an in-house proton treatment-planning system (TPS). While the established PBS dosimetry platform can be applied in a clinical setting, the study is ultimately focused on establishing a PBS proton dosimetry platform for irradiation of large animals under conditions similar to those experienced by astronauts during interplanetary travel.

## MATERIALS AND METHODS

Measurement of individual spot profiles was performed using a gadolinium-based scintillation detector (Lynx^®^, IBA-Dosimetry, Schwarzenbruck, Germany). The Lynx^®^ detector has an active surface area of 300 × 300 mm² with an effective resolution of 0.5 mm. Incoming protons traversing the scintillating screen lose energy via ionization, and this energy is then converted into visible light which is reflected by a mirror and collected by a CCD camera. A 2D ionization chamber array (MatriXX^®^, IBA-Dosimetry, Schwarzenbruck, Germany) was used for measuring profiles from multiple spots. The MatriXX^®^ consists of 1020 parallel-plate ionization chambers, 4.5 mm in diameter by 5 mm high, spaced at 7.6 mm intervals. Although the ionization array detector spacing and resolution are coarse compared with the scintillation detector, the detector spacing and resolution are sufficient for the measurement of profiles from multiple spots, which do not have to change rapidly. The air-filled ionization chambers make the MatriXX^®^ a better choice for dose measurement than a gadolinium-based scintillation detector. Further details on these measurement devices, and their application to PBS dosimetry, have been described by Lin *et al*. [14] and Arjomandy *et al*. [15].

**Fig. 1.**
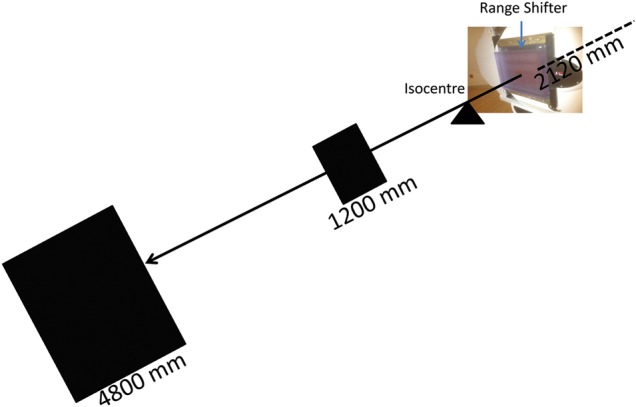
Maximal scanning area (1400 mm SI by 940 mm AP) achievable in pencil beam scanning; the fixed beam is 4800 mm away from the isocentre. The source is at 2120 mm upstream from the isocentre; the 65 mm thick Lucite range shifter is shown at the end of nozzle, and its upstream surface is 410 mm from the isocentre.

The maximum PBS irradiation dimensions at the isocentre are 400 mm and 300 mm along the superior–inferior (SI) and anterior–posterior (AP) directions, respectively. Because an area larger than this is required for large animal irradiation, a PBS technique using an extended SSD was developed in our horizontal (fixed) beam room. The scanning magnets are located at a distance of 1940mm and 2340 mm (averaging 2120 mm) upstream from the isocentre, and they can produce a maximum scan area of 1400 mm (SI) by 940 mm (AP) at the maximum extended distance of 4800 mm, as shown in Fig. 1. In this setup, Yucatan mini pigs were individually irradiated at a distance of 1200 mm beyond the isocentre, corresponding to a scanning area of 580 mm by 320 mm. Beam weights were optimized to provide a uniform total-body dose. In the other set-up, multiple animals were irradiated simultaneously at a distance of 4800 mm, using beam weightings that reproduce the 1989 SPE [5], and which delivers dose preferentially at the surface. The dosimetric characterization of both experimental set-ups is described in this report.

Treatment planning was performed using a commercial PBS TPS for single animal irradiation at 1200 mm (Eclipse v.10.0, Varian Medical Systems, Palo Alto, CA) and an in-house program for animal irradiation at 4800 mm. The principles of PBS treatment planning have been described by Lomax *et al.* [8] and Zhu *et al*. [9].

The in-house TPS program uses the same Bragg peaks as commissioned in the Eclipse TPS, but different spot sizes specific to the extended SSD. As in Eclipse, measured PBS Bragg peaks are similar to the golden beam dataset reported by Clasie *et al.* [10], subject to minor beam line differences between institutions. Previously, Cengel *et al*. [4] simulated the 1989 SPE spectrum using a mixture of 6 MeV and 12 MeV electron beams by matching the FDD. In this work, we matched the proton FDD to that of the mixed electron beams at an entrance surface 4800 mm from the isocentre, using integrated FDD collected during commissioning. The relative weight of each Bragg peak was determined to achieve the desired FDD. In the IBA definition, 1 Monitor Unit (MU) corresponds to 3 nC collected in a 1-cm gap air-filled ion chamber. For a single 225-MeV PBS spot, 1 MU delivers a peak spot dose of 13.2 cGy at the surface and isocentre. The MU required to achieve 1 Gy at 3.5 mm, the inherent buildup thickness of the MatriXX device, was determined for each energy layer. The MU per Gy at 3.5 mm was used to scale the actual MU for each energy layer. Non-uniform spot weighting in the plane perpendicular to the beam direction was determined for each of the involved Bragg peaks to increase the dose at the periphery of the field. The resulting volume irradiated was ∼1400 × 940 × 60 mm^3^.

For single animal irradiation, dose calculations were performed in Eclipse to deliver a uniform whole-body dose of 2 Gy through a total thickness of 220 mm, with the proximal surface located 1200 mm from the isocentre. The resulting volume irradiated was approximately 620 × 320 × 220 mm^3^. Using the MatriXX^®^ detector, quality assurance (QA) measurements were performed at multiple locations perpendicular to the beam direction and at multiple depths (to ensure the depth dose and profiles were delivered as planned).

To increase the transport efficiency for protons below 100 MeV, or ∼75 mm range, from the IBA Proteus cyclotron to the horizontal beam room, a 65-mm thick Lucite RS was installed at the end of the PBS nozzle (Fig. 1). The role of such an RS is to degrade the proton range by 75 mm; therefore, all the required low-energy protons can be achieved by transporting corresponding higher energy protons that have ranges of 75 mm or longer. Introduction of the RS will increase the spot size, however, depending on how far the RS is from the location of interest. Spot characteristics are compared in this study for two RS locations (at nozzle exit and at 70 mm from phantom surface) and two phantom surface locations (1200 mm and 4800 mm downstream of the isocentre). The spot characteristics with the RS located at 70 mm from phantom surface are studied to investigate potential dosimetry improvement beyond current approaches with the RS at the nozzle exit.

## RESULTS

**Fig. 2.**
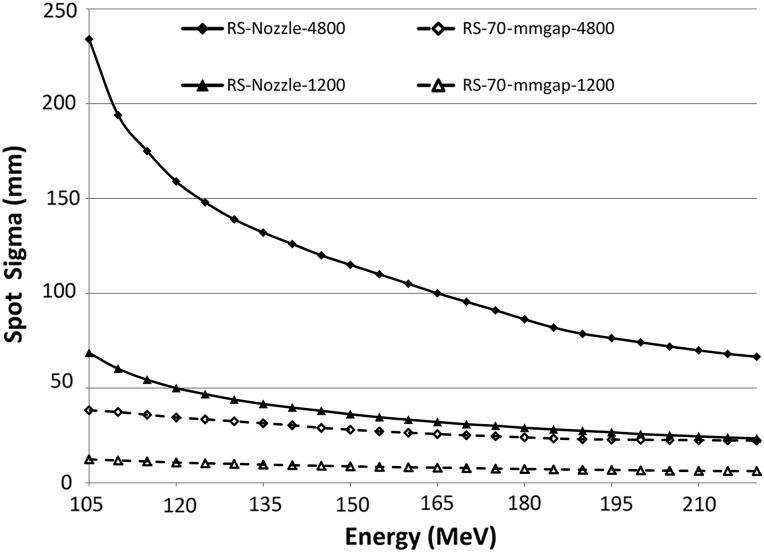
Spot sigma with a range shifter at the nozzle exit and 70 mm in front of the phantom, with the phantom's surface located 1200 and 4800 mm from the isocentre. The 65-mm thick range shifter is made of acrylic and has a 75-mm water-equivalent thickness. The nozzle exit (and range shifter's upstream surface) is 410 mm upstream of the isocentre.

Figure 2 shows the primary spot Gaussian sigma for proton energies from 105 MeV (5 mm range) to 220 MeV (230 mm range) with the RS in place. At 105 MeV, the spot sigma is 234 mm at 4800 mm from the isocentre when the RS is installed at the nozzle, and it drops to 38 mm when the RS is placed 70 mm away from the phantom. With the energy increased to 220 MeV, the spot sigmas are 66 mm and 23 mm at the two RS locations. At a distance of 1200 mm from the isocentre, the Gaussian sigma is 68 mm for 105 MeV and 23 mm for 220 MeV when the RS is located on the nozzle. The spot sigma can be further reduced to 12 mm and 6 mm, respectively, at 1200 mm from the isocentre when the RS is 70 mm from phantom surface. At lower energies, the spot sigma exhibits large differences as a function of distance and the RS position. As proton energy is increased, spot sigmas at 1200 mm with the RS located at the nozzle converge with spot sigmas at 4800 mm with the RS placed 70 mm from the phantom surface.

**Fig. 3.**
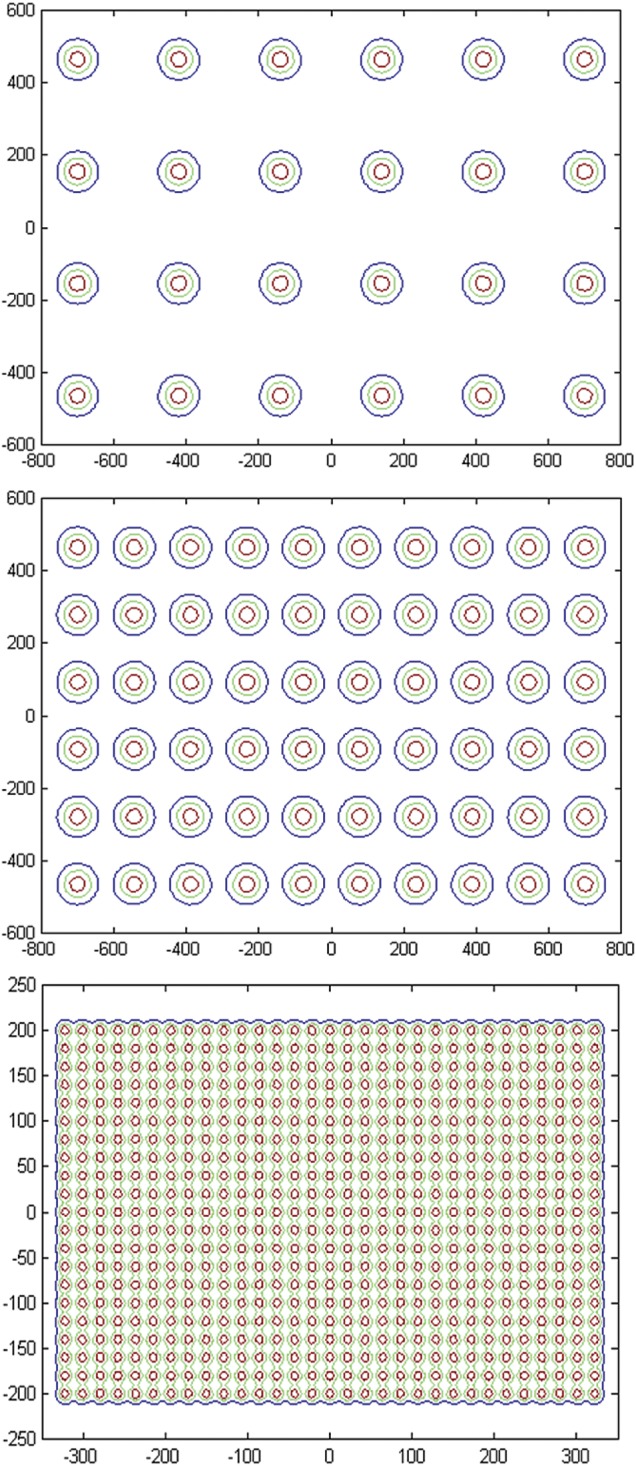
Spot patterns with the range shifter located 70 mm upstream from the phantom surface for: 120-MeV protons and 4800-mm treatment distance (top), 140-MeV protons and 4800-mm treatment distance (middle), and 220-MeV protons and 1200-mm treatment distance (bottom). The isodose lines are 80% (red), 50% (green) and 20% (blue). All units are in mm.

In Fig. 3, the spot sigma data is used to calculate the spot scanning patterns at 4800 mm and 1200 mm from the isocentre when the RS is located 70 mm from the phantom surface. When the phantom surface is 4800 mm from the isocentre, we take advantage of the large spot size and use a 6 × 4 scanning pattern for energies below 125 MeV and a 10 × 6 scanning pattern for energies above 125 MeV. A 31 × 21 pattern is used at 1200 mm from the isocentre, as the Gaussian sigma is smaller and spots need to be closer to each other to achieve dose uniformity. The spot patterns are optimized to minimize the number of spots to reduce radiation delivery time and achieve uniform dose when an RS is used.

**Fig. 4.**
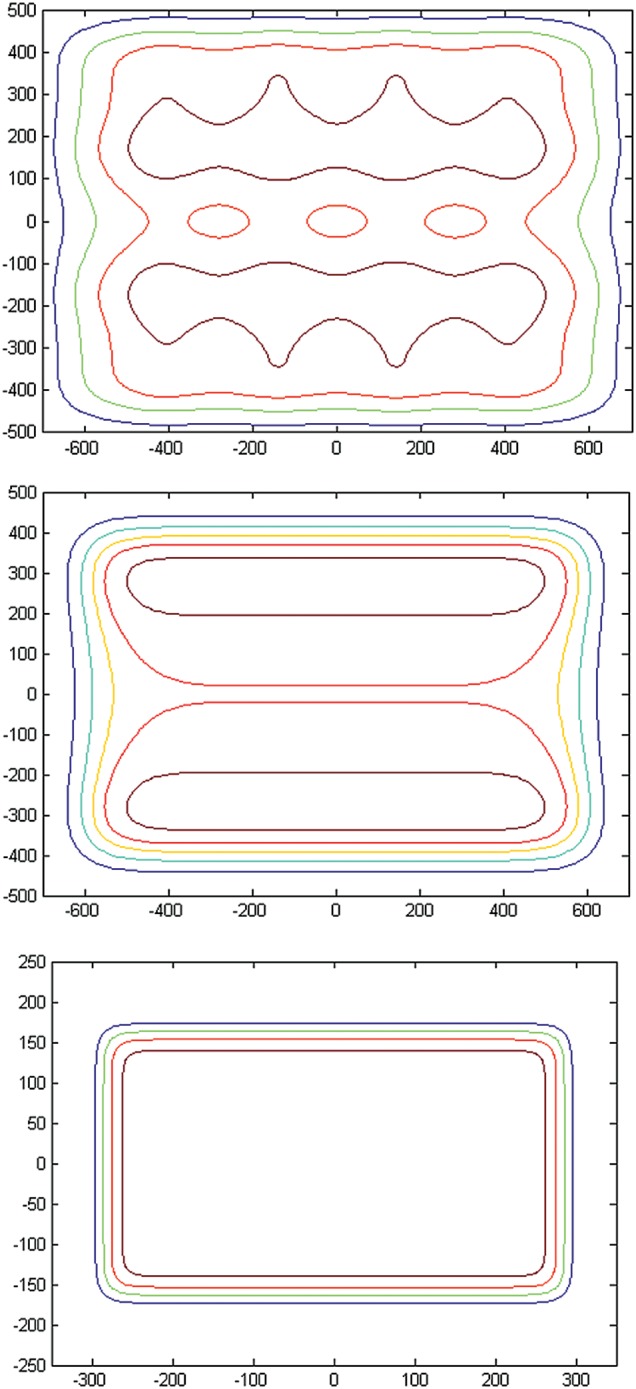
Spot patterns with the range shifter located at the nozzle for: 120-MeV protons and 4800-mm treatment distance (top), 140-MeV protons and 4800-mm treatment distance (middle), and 220-MeV protons and 1200-mm treatment distance (bottom). The isodose lines are 100% (magenta), 98% (red), 95% (green) and 90% (blue). All units are in mm.

Figure 4 shows the corresponding 2D planned surface doses with the RS located at the nozzle. For 120-MeV protons, the optimally weighted 6 × 4 scanning pattern produces a distribution that is within 2% of the desired dose for the central region, but falls to ∼90% at 600 mm and 500 mm in the SI and AP directions, respectively. To achieve a uniform dose over the maximal area of 1400 mm × 940 mm with a marginal dose at 90% of the central uniform dose, non-uniform spot weights are used along the AP direction. For 140-MeV protons, smaller spots in 10 × 6 scanning patterns have a smaller dose falloff margin with similar 2% dose inhomogeneity in the central regions. For treating multiple animals at an extended distance, the resulting dose inhomogeneity is acceptable. Figure 4 also shows the calculated dose distribution from the 31 × 21 scanning pattern for 220-MeV protons at a distance of 1200 mm. In this case, uniform spot weighting achieves 95% of the central dose over a 580 mm (SI) by 320 mm (AP) area, sufficient to treat a single animal. In comparison with that at the surface, the dose distribution at depths is more homogenous because of the larger spot size, and the field size at depths is larger because of the longer distance from the source. In both whole-body and SPE experiments, a large spot size is acceptable, and therefore animal irradiation was performed with the RS located at the exit of the nozzle.

**Fig. 5.**
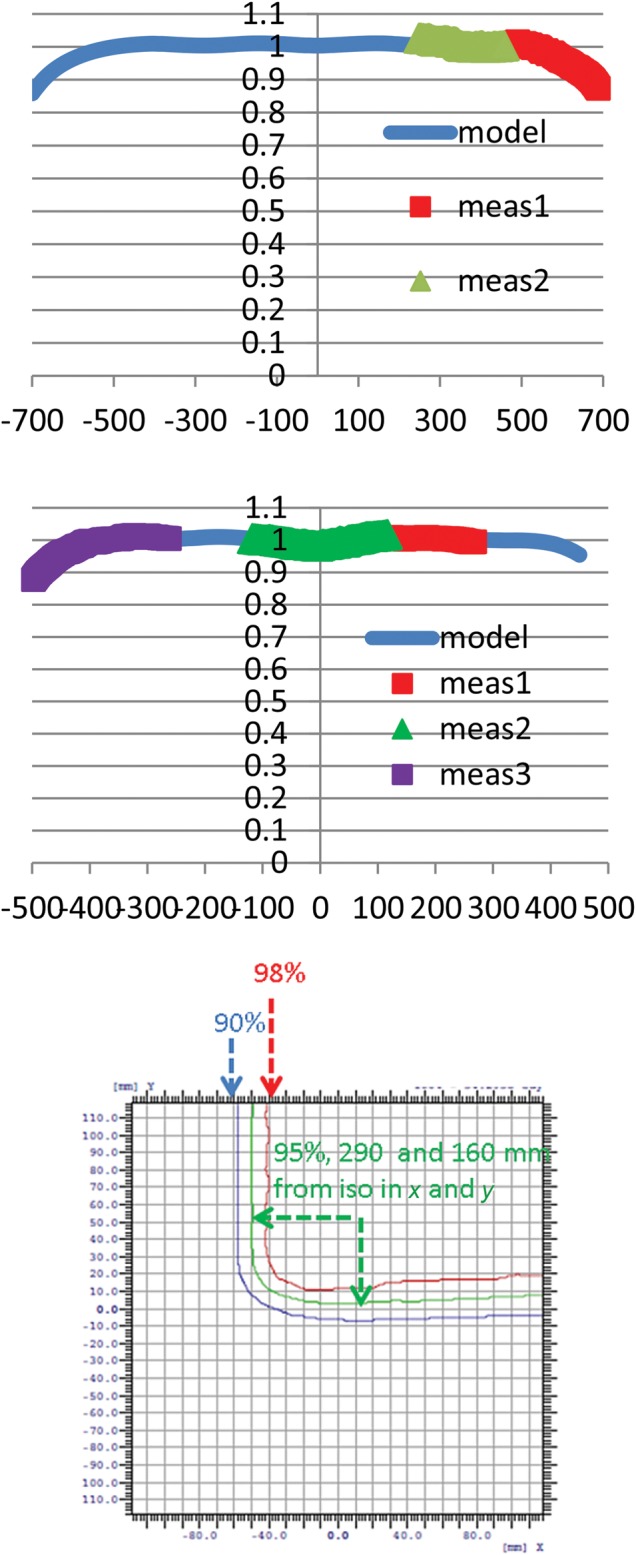
Measurements to validate the relative spot profiles in the SI (top) and the AP (middle) directions with the phantom surface 4800 mm from the isocentre. The measured 2D profile (bottom) is shown for the phantom surface at 1200 mm from the isocentre. All distances are given in mm.

The validation of the dose distribution over a 1400 mm × 940 mm area requires multiple MatriXX measurements as the scanning area is significantly larger than that of the ionization chamber array. Figure 5 shows the central axis comparison of the measured dose profiles along the AP and SI directions, validating the model and the dose uniformity within the central regions and the dose falloff close to the borders. Figure 5 also shows the 2D composite surface dose profile measured at 1200 mm from the isocentre. The 95% isodose is observed at 290 mm (SI) and 160 mm (AP) from the isocentre, further validating the calculation model.

Table 1 lists the sixteen proton energy layers used to match the electron FDD used by Cengel *et al.* [4] to simulate the 1989 SPE. The FDD at 3.5 mm and layer weights are reported for each Bragg peak. The greatest contributions are from 110–117 MeV, corresponding to the range from 12–23 mm. Although not shown in Table 1, 35 proton energy layers are used to achieve uniform whole-body dose to a depth of 220 mm.

**Table 1.** Sixteen layers were used to construct the desired dose distribution

Energy (MeV)	Sigma (mm)	Range (mm)	FDD	weight
105	234	4.7	0.994	0.05
105.5	219	5.3	0.918	0.05
107.5	203	8.3	0.629	0.11
108	201	9.0	0.619	0.04
110	194	11.9	0.519	0.27
112	188	14.9	0.458	0.3
114	180	17.9	0.430	0.29
115	177	19.4	0.415	0.1
117	167	22.5	0.389	0.24
120	159	27.1	0.369	0.07
123	153	31.8	0.355	0.07
126	145	36.6	0.339	0.09
129	141	41.5	0.333	0.08
132	135	46.5	0.320	0.06
135	130	51.7	0.317	0.05
140	127	60.3	0.309	0.02

The FDD (fractional depth dose) of each Bragg peak is at 3.5 mm for the IBA MatriXX effective water-equivalent thickness. Dose weight refers to dose at the peak, i.e. 0.05 means 5 cGy at the peak of 105 MeV per Gy at 3.5 mm.

**Fig. 6.**
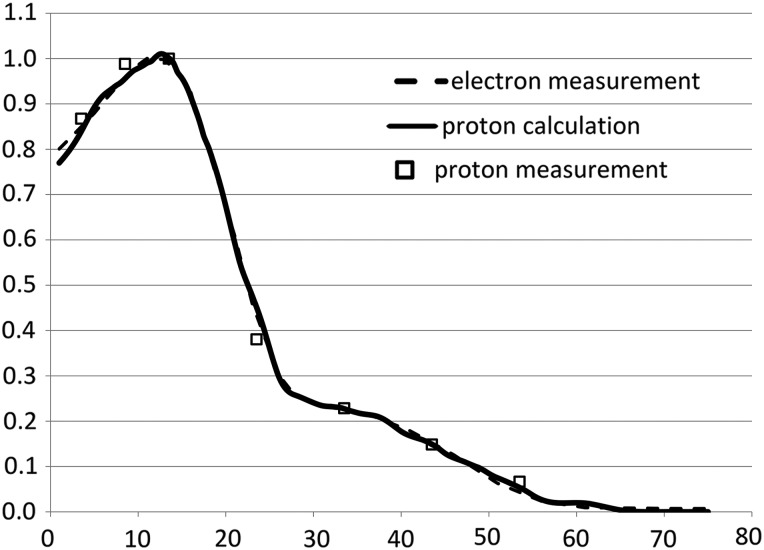
Measurements to validate the fractional depth dose in the phantom. The proton depth dose is designed to match the electron depth dose (dashed thin line), which is a mixture of 6 MeV and 12 MeV to simulate the 1989 SPE's proton depth dose. The solid thick line is from calculation, and the square markers are from the measurement of the proton depth dose.

Figure 6 shows proton measurements superimposed on the planned proton and mixed electron beam depth doses used to simulate the 1989 SPE event (treatment distance = 4800 mm from isocentre). In all cases, measurements agree with the calculation to within 2%. For total-body irradiation at 1200 mm from the isocentre, similar measurements performed at depths ranging from the surface to 220 mm demonstrate < 3% difference compared with the calculation.

## DISCUSSION

To investigate the effects of SPE irradiation *in vivo*, Coutrakon *et al.* [16] at Loma Linda University and Medical Center used a PBS technique and the proton spectrum of the 1989 SPE to irradiate mice. Animals were irradiated in an experimental room using a 3 × 3 spot pattern covering ∼1 m^2^. The SPE spectrum was modeled using 18 weighted proton energies spanning a range from 30–210 MeV. Dosimetry was performed using a large parallel plate chamber and thermoluminescent dosimeters (TLDs) attached to the cages. The group performed subsequent experiments evaluating liver response and hematopoetic effects [17, 18]. Due to the lack of a large animal proton irradiation platform, Cengel *et al*. initially used a mixture of 6-MeV and 12-MeV eletron beams to approximate the 1989 SPE proton spectrum in the irradiation of Yucatan minipigs [4]. However, as different particle interaction mechanisms are involved in dose deposition, matching the depth dose of the 1989 SPE proton spectrum with a mixture of electron beams does not accurately represent the real safety hazard faced by astronauts. In this study, we have established a dosimetry platform for PBS proton irradiation of large animals and reproduced the experimental conditions of the Cengel study, using spot scanning with optimally weighted spots at an extended distance (4800 mm from the isocentre).

Further, we describe a technique for uniform total-body irradiation of large animals using PBS. In both scenarios, the spot Gaussian sigma is larger than 23 mm with the RS located in the nozzle, minimizing any interplay effect between PBS delivery and respiratory motion. PBS spots are scanned at between 20 and 40 m/s, and depending on proton energy and the distance between adjacent spots, it takes from 1–80 ms for proton spots to settle in the scanning positions. It is important to note that these irradiations are typically performed over several hours with multiple paintings to simulate real exposure conditions. Although the scanning speed and settling time between adjacent spots are relatively short, mutiple energy layers and a large MU over a big area are needed; thus each painting requires ∼10 min to deliver all of the energies in order to deliver the 0.25 Gy maximal dose to each side of the animal. To avoid the medical risk and potential confounding of results from prolonged anesthesia, it is necessary to irradiate the animals while they are awake and have the ability to access nutritional support in the irradiation enclosure [5–6, 19]. These enclosures allow modest animal movement in the SI and AP dimensions within the confines of the enclosure, where the field is homogeneous. However, the lateral mobility is limited so that the animal cannot move about this axis (where the dose is inhomogeneous), i.e. turn around. This has raised concerns as to whether residual animal movement and internal organ movement could potentially have an important impact on the accurate delivery of the intended dose distribution using PBS techniques [20]. By keeping the RS at the nozzle, the large spot sigma (23 mm) for the highest energy (220 MeV) makes the treatment plans more robust for animal motion during irradiation and minimizes this concern. However, larger spots at lower energies and longer SSDs might compromise ability to vary the dose distribution in the AP and SI dimensions. To treat large animals with dose distributions intended to simulate the steeper dose gradients, such as in the intensity-modulated proton therapy used in typical patient treatments, spot size can be reduced by placing the RS <70 mm from the surface of the animals, allowing spot sigmas below 6 mm. In this case, internal and external movement will need to be addressed, but given the typically short duration of patient treatments, anesthesia can be used more safely.

## CONCLUSION

The newly established PBS dosimetry platform is valid for irradiation of multiple animals using a spectrum that simulates SPEs, as well as for uniform whole-body irradiation of single animals. Measurements compare favourably with simulations over a broad range of irradiation conditions, proton energies, and PBS spot characteristics.

## FUNDING

This work was supported by the Center of Acute Radiation Research (CARR) grant from the National Space Biomedical Research Institute (NSBRI) through NASA NCC 9–58, NIH Training Grant 2T32CA00967 and NIH CA-140116. Olivier De Wilde from Research and Development, Ion Beam Applications (Louvain-la-Neuve, Belgium), kindly provided the specification of PBS scanning speed and settlement time involved in spot scanning.
